# Benchmarking diffusion models against state-of-the-art architectures for OCT fluid biomarker segmentation

**DOI:** 10.1371/journal.pone.0335615

**Published:** 2025-10-29

**Authors:** Katherine Du, Utkarsh Doshi, Benjamin DiCenzo, Jessica Jiang, Ethan Wu, Adarsh Gadari, Sharat Chandra Vupparaboina, Elham Sadeghi, Sandeep Chandra Bollepalli, José-Alain Sahel, Jay Chhablani, Kiran Kumar Vupparaboina

**Affiliations:** Department of Ophthalmology, University of Pittsburgh Medical Center, Pittsburgh, Pennsylvania, United States of America; University of Warmia, POLAND

## Abstract

**Objectives:**

Retinal diseases, major causes of vision impairment and blindness, are assessed using optical coherence tomography (OCT) scans. Automated report generation for retinal OCT scans, powered by deep learning, can help standardize interpretations and track retinal disease in clinical settings. A key challenge is accurately segmenting retinal disease signatures. This study explores using the diffusion model to segment subretinal fluid (SRF), intraretinal fluid (IRF), and pigment epithelial detachment (PED) in typical clinical settings, comparing their performance to other leading segmentation models.

**Methods:**

We labeled OCT scans and extracted those with specific pathologic retinal features: 269 scans with SRF, 224 scans with IRF, and 114 scans with PED. Three trained reviewers manually segmented these features for downstream analysis. Using manually segmented scans as the ground truth, we trained the diffusion model, Nested U-Net, nnU-Net, TransUNet, and SwinUNet to predict these segmentations. All models were evaluated using 5-fold cross-validation, with performance measured by Dice coefficient, sensitivity, specificity, Pearson correlation coefficient, and R^2^.

**Results:**

All models show high similarly with ground truth segmentations in predicting SRF, IRF, and PED, as shown by the Dice coefficient (Diffusion model: 0.81 ± 0.12, 0.66 ± 0.09, 0.75** **± 0.11). The diffusion model has relatively higher sensitivity compared to most other models, while all models display very high specificity. The Pearson correlation coefficient and R^2^ values show strongly associated pixel quantification of segmented areas for models, with the nnU-Net model performing the strongest overall.

**Conclusion:**

This study demonstrates that while diffusion models can comparably segment retinal pathologies using a limited number of manually annotated scans, the nnU-Net model remains the most effective overall for automated OCT analysis.

## Introduction

Retinal diseases, including age-related macular degeneration (AMD), diabetic retinopathy (DR), and central serous chorioretinopathy (CSCR) are major contributors to vision impairment and blindness [1]. With the growth of deep learning in medical imaging, a potential application in ophthalmology is automated report generation for optical coherence tomography (OCT) scans, the key imaging modality for evaluating retinal diseases which provides detailed cross-sectional images of posterior segment structures including retinal layers [2–3]. The creation of these automated reports as a medical decision support tool can facilitate standardized interpretations and reporting of retinal disease status in clinic settings [4–6]. It also has the potential to increase the accessibility of ophthalmologic care for patients in areas with shortages, by providing a retinal OCT scan report to identify pathologic retinal features and triaging the patient to higher levels of care if necessary [7–8]. Additionally, machine learning techniques can be applied to track the progression over time of the overall state of the retina or of specific pathologic features via qualitative and quantitative metrics [9–10].

The segmentation of specific retinal pathologies, including subretinal fluid (SRF), intraretinal fluid (IRF), pigment epithelial detachment (PED), drusen, and hyperreflective dots or foci is a crucial step in advancing automated report generation for OCT scans. In the recent past, various machine learning (ML) based approaches have been fairly accurate in segmenting several key disease features, including SRF, IRF and PED [11–12]. These approaches predominantly involve convolutional neural network (CNN)-based models, including U-Net and encoder-decoder fully convolutional neural networks (FCNNs). For instance, a study which employed the U-Net model to segment five crucial neovascular age-related macular degeneration (nAMD) features resulted in high correlation between automated and manual segmentations and moderate Dice scores [13]. Other studies which use an encoder-decoder style FCNN demonstrate its applicability in detecting and quantifying macular fluid and PED in conventional OCT images [14–16]. Transformer-based segmentation models, such as Swin-UNet and TransUNet, have also been demonstrated to effectively segment SRF, IRF, and PED [17–18]. However, these approaches have been evaluated primarily on disparate controlled datasets, leaving their generalizability to unseen data uncertain.

Diffusion models are a class of generative models that have gained significant attention in recent years for their ability to generate high-quality images through a stepwise denoising process [19]. These models work by progressively adding noise to data and then learning to reverse this process, iteratively recovering the original data. In the context of OCT image analysis, diffusion models may be well suited for tasks such as segmentation and mask generation [20–21]. Retinal OCT images often present challenges due to noise, low contrast, and variability in the tissue structures, and diffusion models have been shown to enhance scan quality [22–24]. However, there is sparse literature on their potential for improving segmentation in medical imaging tasks, where precision is critical.

The objective of this study is to evaluate the effectiveness of diffusion models in segmenting common retinal disease features, specifically SRF, IRF, and PED. We aim to evaluate diffusion models against benchmark approaches in a practical setting, using a relatively small set of manually annotated retinal biomarkers—an amount feasible for clinical centers given the substantial time, effort, and expertise required. Our goal is to identify which machine learning model is most suitable for retinal pathology segmentation under typical resource limitations. The OCT scans utilized include dry AMD, wet AMD, DR, and CSCR eyes. We employ a custom retinal biomarker segmentation tool for the manual segmentation of these retinal pathologies and automated preparation of the scans for downstream analysis. Using manually segmented scans as the ground truth, we train the diffusion model to segment SRF, IRF and PED. We compare the proposed approach vis-a-vis segmentation based on the nnU-Net, TransUNet, Swin-UNet, and Nested U-Net models. This study provides a stepping stone to enhancing automated OCT analysis by introducing the diffusion model as an alternative for robustly segmenting retinal pathologies.

## Methods

### Dataset

This retrospective study was conducted in accordance with the Declaration of Helsinki, and was approved by the Institutional Review Board of the University of Pittsburgh Medical Center (Study ID: STUDY20030263; *Retrospective Study of Presentations & Outcome of Vitreo-Retinal Diseases* (). Written informed consent was obtained from all participants for the inclusion of their retrospective data. All OCT data were de-identified prior to analysis by replacing patient medical record numbers with random numbers and excluding any demographic information; authors did not have access to patient identifiers during or after data collection.

We utilized OCT volumes obtained from the Cirrus 5000 OCT device (Carl Zeiss Meditec) from 200 unique eyes corresponding to individual subjects diagnosed with dry age-related macular degeneration (AMD), wet AMD, diabetic retinopathy (DR), or central serous chorioretinopathy (CSCR)*.* The dataset was roughly balanced, with about one-third of scans from each condition. Each Cirrus OCT volume consisted of 128 B-scans, with a lateral resolution of 6 mm and a depth resolution of 2 mm, (512 × 1024 pixels). From each volume,16 B-scans were uniformly sampled, yielding 3,200 B-scans in total. A subset of these scans containing retinal pathologies was manually segmented*.* Patients were screened between 2017 and 2019, and the anonymized dataset was accessed on April 8 2024 for research purposes.

### Feature description

**Fig 1:**
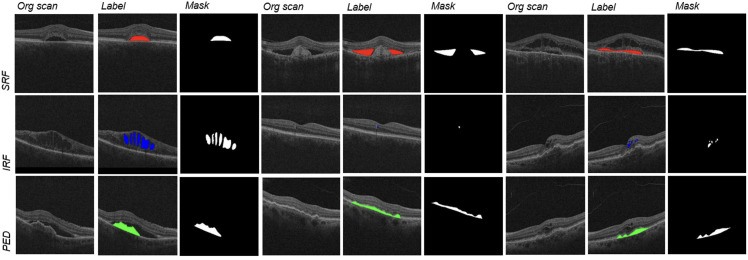
Examples of OCT scan pathological feature masks used as ground truth labels for automated prediction and segmentation of these pathologies by machine learning models.

The various retinal disease signatures segmented for training and testing the diffusion model include subretinal fluid, intraretinal fluid, and pigment epithelial detachment (Fig 1).

### Collect scans with disease features

To this end, we leveraged our previously in-house developed OCT image-labeling software to annotate retinal features qualitatively including scan quality, reason for poor quality scan (if applicable), healthy/diseased scan, foveal scan, drusen, SRF*,* IRF*,* PED, geographic atrophy, hyperreflective dots, and hyperreflective foci [25]. Next, using these qualitative labels, we filtered out OCT scans with SRF, IRF and PED features for segmentation*.* The scans segmented include those from dry AMD, wet AMD, diabetic retinopathy, and CSCR eyes. Table 1 shows the counts of pathologic features segmented.

**Table 1:** Counts of pathologic features segmented on OCT scans for each retinal disease subtype and in total.

	Dry AMD	Wet AMD	DR	CSCR	Total
**SRF**	0	54	7	208	269
**IRF**	0	10	180	34	224
**PED**	12	60	13	29	114

### Annotation strategy

Three trained reviewers independently segmented the OCT B-scan features under consideration using an in-house retinal biomarkers segmentation tool, with guidance from a retinal ophthalmologist. To ensure that multiple reviewers can label scan features in a standardized manner, the concurrence between reviewers and the retinal ophthalmologist was established beforehand. At the end of the segmentation process, the retinal ophthalmologist reviewed all the segmentations to ensure their accuracy.

### Models

#### Nested U-Net model.

The Nested U-Net is an advanced variant of the standard U-Net architecture, designed for precise medical image segmentation [26]. It features a symmetric encoder–decoder structure with dense skip connections and residual blocks across multiple depth levels, enhancing multi-scale feature fusion. Residual Dense Blocks mitigate gradient vanishing and improve feature reuse, while the dense skip connections preserve fine-grained spatial information, allowing the network to capture both local and global context effectively.

#### nnU-Net model.

nnU-Net is a self-adapting U-Net–based framework for biomedical image segmentation [27]. Unlike manually designed architectures, nnU-Net automatically configures preprocessing, network architecture, training, and post-processing steps to match the properties of a given dataset, serving as a strong and standardized baseline across diverse medical imaging tasks.

#### TransUNet model.

TransUNet is a hybrid architecture that integrates convolutional and transformer components for medical image segmentation [28]. Its encoder consists of a CNN backbone for multi-scale feature extraction, while the deepest features are processed by a Vision Transformer to capture global dependencies. Skip connections are drawn from the CNN encoder and fused into a U-Net–style decoder. By combining the global context modeling of transformers with the local detail preservation of CNN skip connections, TransUNet achieves robust segmentation performance.

#### Swin-UNet model.

Swin-UNet is a transformer-based extension of U-Net that replaces traditional convolutional layers with Swin Transformer blocks [29]. It employs hierarchical feature extraction and shifted window self-attention to capture both local and global context efficiently. Structured in an encoder–decoder fashion similar to U-Net, Swin-UNet preserves fine-grained spatial details while maintaining long-range dependencies, making it well suited for dense prediction tasks.

#### Diffusion model.

Diffusion models are a class of generative models that learn to produce data by reversing a gradual forward noising process [19,30,31]. They operate by adding Gaussian noise to training data in a stepwise fashion and then learning to reconstruct the original data by inverting this process. Once trained, these models can generate new data by applying the learned denoising steps to random noise.

For this study, we utilized MedSegDiff [32], a diffusion probabilistic model (DPM) tailored for medical image segmentation. MedSegDiff introduces two key innovations:

1. Dynamic Conditional Encoding: modulates feature conditions at each denoising step to prioritize region-specific attention, addressing spatial heterogeneity in medical images
2. Feature Frequency Parser (FF-Parser): suppresses high-frequency noise during reverse diffusion while preserving structural details

### Model training

In this study, we conducted a comprehensive comparative analysis of the diffusion model and four other state-of-the-art architectures for the segmentation of three distinct retinal biomarkers: SRF, IRF, and PED. All models were trained on NVIDIA GTX 2080 GPUs (12 GB VRAM) using 5-fold cross-validation. OCT images were resized to 256 × 256 pixels, and stratification was applied to ensure that scans from the same subject appeared only in either the training or test set.

- **Nested U-Net:** trained for up to 200 epochs with a learning rate of 1e-4, reduced by a factor of 2 every 8 epochs. Batch size was 4. Early stopping was applied with a patience of 25 epochs. Loss: Dice + Binary Cross-Entropy.
- **nnU-Net:** trained for 1000 epochs with a linearly decaying learning rate reaching 0 at the final epoch. Loss: Dice + Cross-Entropy.
- **TransUNet:** initialized from pretrained checkpoints, trained for up to 200 epochs with a learning rate of 1e-4 and weight decay of 1e-5. Early stopping with patience 50 was applied. Training converged after 165–200 epochs. Loss: Dice + Binary Cross-Entropy.
- **Swin-UNet:** initialized from pretrained checkpoints, trained for up to 200 epochs with a learning rate of 1e-4 and weight decay of 1e-5. Early stopping with patience 50 was applied. Training converged after 95–130 epochs. Loss: Dice + Binary Cross-Entropy. Due to its architecture, Swin-UNet input image size is 224x224 pixels. Images were resized to 256x256 pixels for Dice score calculation.
- **Diffusion (MedSegDiff):** trained uniformly for 500 epochs with a fixed learning rate of 1e-4 and batch size 4. Loss: Mean Squared Error.

### Evaluation metrics

For each of the retinal biomarkers segmented, we evaluated the performance of the diffusion and nested U-Net models using the following evaluation metrics:

**Dice similarity coefficient:** The Dice coefficient (DSC) quantifies the spatial overlap between the predicted and ground truth segmentation [33]*.* It ranges from 0, indicating no overlap, to 1, indicating perfect overlap. This metric provides a balanced assessment of segmentation performance by incorporating both precision and recall, making it particularly suitable for medical image analysis.

**Sensitivity:** Sensitivity measures the model’s ability to correctly identify positive cases [34]. It is defined as the proportion of true positives among all actual positive instances.

**Specificity:** Specificity evaluates the model’s ability to correctly identify negative cases [34]. It represents the proportion of true negatives among all actual negative instances.

**Correlation coefficient and R**^**2**^: The correlation coefficient quantifies the linear relationship between two variables, ranging from −1 indicating perfect negative correlation to +1 indicating perfect positive correlation, with 0 indicating no correlation [35]*.* The Pearson correlation coefficient (PCC) measures the strength and direction of the linear relationship between two variables, while R² represents the proportion of the variance in one variable that is explained by the linear relationship with the other variable [35].

## Results

**Fig 2:**
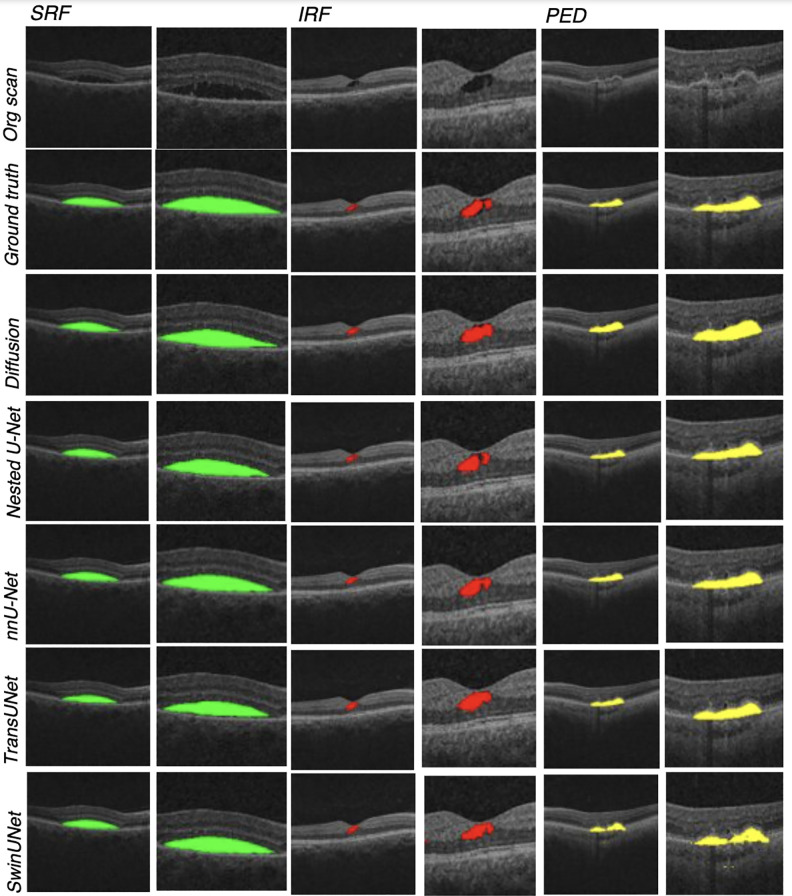
Examples of OCT scans manually segmented for each retinal biomarker in the ground truth segmentations, followed by the predicted segmentations from the five deep learning models.

For the nested U-Net, nnU-Net, TransUNet, SwinUNet, and diffusion models, the DSC conveys high similarity between the manually labeled ground truth segmentations and the ones predicted by each model for identifying SRF, IRF, and PED. As shown in Fig 2, the predictions made by the models were similar to the ground truth segmentations for the majority of OCT scans. However, the diffusion models display slightly lower DSCs compared to the other model types overall, except for surpassing SwinUNet in IRF and PED (Table 2). The nnU-Net provides the most accurate segmentations of the retinal biomarkers out of all the models. Specifically, the models are best at predicting SRF and show comparable abilities to correctly segment the feature, with DSCs ranging from 0.81 ± 0.12 for the diffusion model to 0.86 ± 0.10 for the nnU-Net model. For IRF, the nnU-Net’s predictions align best with the ground truth segmentations (DSC: 0.72 ± 0.10) and the SwinUNet model the least (DSC: 0.65** **± 0.09). Lastly, the average predictions for all models were relatively robust for PED (range of DSCs from 0.85** **± 0.09 for nnU-Net model to 0.73 ± 0.10 for SwinUNet model).

**Table 2:** Dice coefficients of the five deep learning models for the segmentation of OCT scan pathology using 5-fold cross-validation.

	SRF	IRF	PED
**Nested U-Net**	0.85 ± 0.10	0.70 ± 0.10	0.82 ± 0.10
**nnU-Net**	0.86 ± 0.10	0.72 ± 0.10	0.85 ± 0.09
**TransUNet**	0.85 ± 0.10	0.67 ± 0.10	0.83 ± 0.09
**SwinUNet**	0.83 ± 0.11	0.65 ± 0.09	0.73 ± 0.10
**Diffusion**	0.81 ± 0.12	0.66 ± 0.09	0.75 ± 0.11

The sensitivity and specificity of the diffusion and other models, in comparison to the ground truth, vary depending on the retinal features that are segmented, as shown in Table 3. The diffusion model displays relatively increased sensitivity in segmenting SRF, IRF, and PED. Sensitivity measures how well the models correctly identify and segment all relevant regions (true positives) that should be segmented. Hence, all five models successfully identify and segment most of the desired regions, with diffusion models ranking second or third among the compared models. The specificity is also robust for all five models across all retinal features, indicating that the models are proficient at identifying regions that should not be segmented (true negatives).

**Table 3:** Evaluation metrics of the five deep learning models for the segmentation of OCT scan pathology using 5-fold cross-validation.

	Nested U-Net	nnU-Net	TransUNet	SwinUNet	Diffusion
***SRF***					
**Sensitivity**	0.84 ± 0.13	0.86 ± 0.13	0.85 ± 0.13	0.84 ± 0.13	0.85 ± 0.15
**Specificity**	1.00 ± 0.00	1.00 ± 0.00	1.00 ± 0.00	1.00 ± 0.00	1.00 ± 0.01
**Pearson correlation**	0.86 ± 0.09	0.86 ± 0.09	0.85 ± 0.10	0.83 ± 0.10	0.82 ± 0.11
**R** ^**2**^	0.71 ± 0.19	0.71 ± 0.22	0.70 ± 0.24	0.63 ± 0.24	0.59 ± 0.30
***IRF***					
**Sensitivity**	0.68 ± 0.14	0.70 ± 0.14	0.77 ± 0.14	0.70 ± 0.14	0.73 ± 0.15
**Specificity**	1.00 ± 0.01	1.00 ± 0.00	1.00 ± 0.00	1.00 ± 0.00	1.00 ± 0.00
**Pearson correlation**	0.70 ± 0.10	0.72 ± 0.10	0.68 ± 0.10	0.66 ± 0.08	0.67 ± 0.08
**R** ^**2**^	0.41 ± 0.21	0.43 ± 0.25	0.22 ± 0.33	0.24 ± 0.27	0.24 ± 0.29
***PED***					
**Sensitivity**	0.81 ± 0.14	0.87 ± 0.11	0.85 ± 0.11	0.74 ± 0.16	0.84 ± 0.16
**Specificity**	1.00 ± 0.00	1.00 ± 0.00	1.00 ± 0.00	1.00 ± 0.00	1.00 ± 0.00
**Pearson correlation**	0.83 ± 0.09	0.85 ± 0.09	0.84 ± 0.08	0.74 ± 0.10	0.76 ± 0.10
**R** ^**2**^	0.64 ± 0.24	0.67 ± 0.23	0.65 ± 0.23	0.45 ± 0.24	0.41 ± 0.38

**Fig 3:**
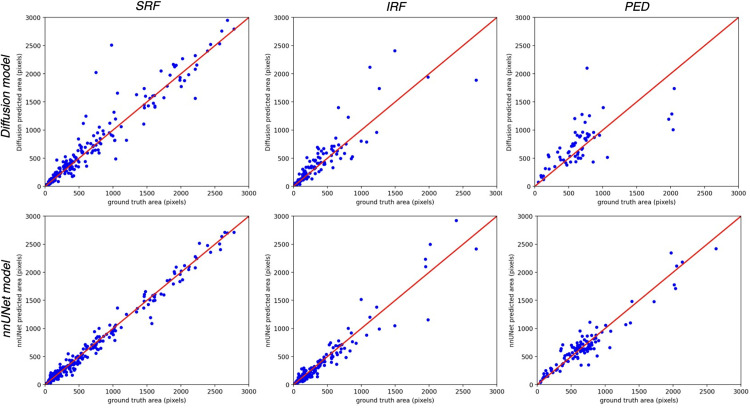
Correlation of areas segmented manually (ground truth) versus areas segmented by diffusion and nnU-Net models (predicted area from strongest compared model) per OCT scan.

Lastly, in quantifying pixel area between ground truth and predicted segmentations, the nnU-Net model shows a slightly improved PCC and R^2^ for SRF, IRF, and PED (Table 3). The PCC is between 0.67–0.82 for the diffusion model and between 0.72–0.86 for the nnU-Net model for all features segmented. The R^2^ values are between 0.24–0.59 for the diffusion model and between 0.43–0.71 for the nnU-Net model. Hence, there is a moderate to strong linear relationship and moderate explained variability between the ground truth and predicted segmentation pixel areas for all models; in other words, the quantified segmentation areas show a moderate to strong association between the ground truth and predicted segmentations. The models are able to adequately quantify both smaller and larger areas of SRF, IRF, and PED compared to ground truth segmentations (Fig 3).

## Discussion

Our study demonstrates that the diffusion model is a novel machine learning technique that can be applied to detect pathologic retinal features with comparable accuracies to other state-of-the-art machine learning models. On our dataset, the diffusion models display slightly lower DSCs overall, while the nnU-Net consistently achieves the highest performance across all three retinal biomarkers. The sensitivity and specificity metrics demonstrate that the diffusion model is more inclusive of pathologic retinal feature areas compared to most other models, and all models are superior in their ability to avoid segmenting the image background. Moreover, the segmentation areas predicted by the diffusion and other models are moderately correlated with the ground truth areas, with the nnU-Net model exhibiting the highest PCC and R^2^ values for fluid and PED.

Nested U-Net, nnU-Net, TransUNet, and SwinUNet are advanced deep learning archetectures built upon or inspired by the original U-Net, commonly used for medical image segmentation [36]. They have been shown to effectively segment retinal biomarkers in several studies. On the other hand, a diffusion model generates images by iteratively refining noisy inputs, reversing a diffusion process that progressively adds noise to the image [32]. For the task of segmenting scans, the diffusion model gradually denoises a corrupted image and can generate the segmentation, but it usually requires more computation and training compared to the U-Net-based models which directly learns to predict missing pixels. However, if trained thoroughly, diffusion models may be able to produce more realistic and coherent results by leveraging their generative properties.

**Fig 4:**
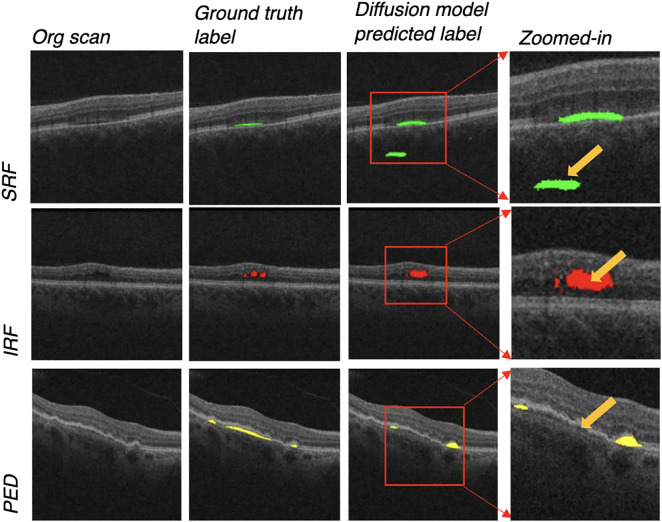
Examples of OCT scans for each retinal biomarker which presented challenges for the diffusion model in achieving complete segmentation accuracy.

While the diffusion model and various U-Net-based models show similar performance, due to the relatively small size of our segmented scans dataset used in training and testing the models – with 269 total scans for SRF, 224 scans for IRF, and 114 scans for PED – we can identify some potential challenges for the diffusion model in segmenting these retinal biomarkers to accurately quantify their areas [37]*.* The diffusion model inherently requires more input data to build a robust model because of the nature of its learning and generative processes [38]. However, we aimed to balance this with the practical limitations for many clinical centers to manually segment OCT scans due to the substantial human time, effort, and expertise needed, as our goal is to evaluate the diffusion model against other model types for real-world use. Hence, for a few predictions, some of the output OCT scans from the diffusion model had regions segmented outside of the retinal tissue layers and in the background (Fig 4, row of SRF). This may be due to textural differences in the background, with small patches of solid black being segmented as it resembles fluid texture compared to the typical speckled gray background*.* Additionally, artifacts within the retinal layers themselves such as the black shadowing in the IRF scan of Fig 4 caused the predicted segmentation to be slightly larger than that of the ground truth scan but still representative overall. Lastly, the thin nature of some double-layer sign PEDs resulted in the segmentation predictions of the diffusion model to cover a portion of the retinal pathology but not the full feature (Fig 4, row of PED).

Limitations of this study include the segmentation of larger retinal pathologies but not significantly smaller features, such as drusen, hyperreflective dots, and hyperreflective foci*.* Future studies can compare the ability of the diffusion model and U-Net-based models in segmenting these retinal biomarkers and others including geographic atrophy. This could help elucidate whether these machine learning models show differences in correctly distinguishing and segmenting smaller areas or areas outside of the retinal tissue layers, as well as how the performance compares between various models. Another limitation is the sample size of the segmented features, as diffusion models tend to produce highly accurate representations with more extensive training data [39]*.* Although we started with thousands of labeled OCT scans, the segmented features represented only a small proportion of the dataset, and we selected examples with variability and good quality, limiting our sample size.

In conclusion, this study evaluates diffusion models as an alternative to conventional U-Net-based approaches for OCT retinal pathology segmentation, showing their potential under typical clinical data limitations. While all models demonstrate robust performance in segmentation of these features, the diffusion model technique shows relatively greater sensitivity overall. However, under realistic clinical constraints involving limited annotated data, the diffusion model demonstrates limited segmentation accuracy and area correlations compared to the other state-of-the-art models such as nnU-Net. From a broader outlook, these models can be applied in the process of automated report generation of retinal OCT scans to identify the regions of pathologic retinal features. As shown by this study, a crucial application of these models is to accurately quantify pathologic retinal feature areas to personalize patient care*.* Quantifying segmentation area will aid in tracking the progression of retinal pathology over time, during its natural course or after ophthalmological treatments to gauge treatment efficacy. This will support the development of teleophthalmology for structured reporting in the clinic as well as promote the accessibility of retinal diagnostic imaging for underserved communities.
